# Giant Parathyroid Adenoma: A Case Report

**DOI:** 10.7759/cureus.34140

**Published:** 2023-01-24

**Authors:** Siddharth Shah, Priscilla Fujikawa, Kenneth Brand, Viraj Munshi, Kashyap Patel

**Affiliations:** 1 Internal Medicine, Lewis Gale Medical Center, Salem, USA; 2 Internal Medicine, Edward Via College of Osteopathic Medicine, Blacksburg, USA

**Keywords:** parathyroid and lipid disorders, hungry bone syndrome, parathyroid function test, parathyroid hormone (pth), postoperative parathyroid hormone (pth), giant parathyroid adenoma, parathyroid gland adenoma

## Abstract

Parathyroid adenomas rarely weigh more than 4 grams. Our patient had a 5.3-gram adenoma causing bilateral knee pain limiting mobility, constipation, low back pain, and frontal headache. Presenting with calcium of greater than 17 mg/dl, the patient was treated with two rounds of hemodialysis, calcitonin, Zoledronate, and aggressive IV hydration to decrease calcium levels before parathyroidectomy. The patient then went on to develop the hungry bone syndrome, which was treated with calcium carbonate and calcitriol. This rare giant parathyroid adenoma presents a unique opportunity to learn about the pathogenesis and treatment of longstanding hyperparathyroidism causing hypercalcemia-associated symptoms and hungry bone syndrome after parathyroidectomy.

## Introduction

Parathyroid adenomas are responsible for 85% of hyperparathyroidism and tend to be <2cm in size and <1gram in weight [1]. Large parathyroid adenomas between 2-4 grams are in the upper 95^th^ percentile [2-4]. Although parathyroid adenomas are common in postmenopausal women, giant parathyroid adenomas tend to be more common in men [1,5]. This rare parathyroid adenoma falls in the top one percentile by weight and size, resulting in delayed onset of symptoms and eventual hungry bone syndrome post parathyroidectomy.

## Case presentation

This patient was a 64-year-old male with a history of hypertension and diabetes who presented with the inability to walk for nine days due to 7 out of 10 dull, bilateral knee pain radiating to shins. Additionally, he was constipated for five days and had a frontal headache and non-radiating low back pain for three weeks. The night before admission, he fell when trying to get up due to severe knee pain without loss of consciousness or head trauma.

On admission, X-rays of the knee and lumbar spine were unremarkable, but the lab workup showed elevated ionized calcium, intact parathyroid hormone, creatinine, and phosphorus (Table 1).

**Table 1:** Laboratory Values

	Values	Reference Range
Intact Parathyroid Hormone	>2,000 pg/ml	18.5-88 pg/mL
Creatinine	5.7 mg/dL	0.74-1.35 mg/dL
Phosphorous	6.3 mg/dL	2.5-4.9 mg/dL
Ionized Calcium	2.22 mmol/L	1.15-1.35 mmol/L

**Figure 1:**
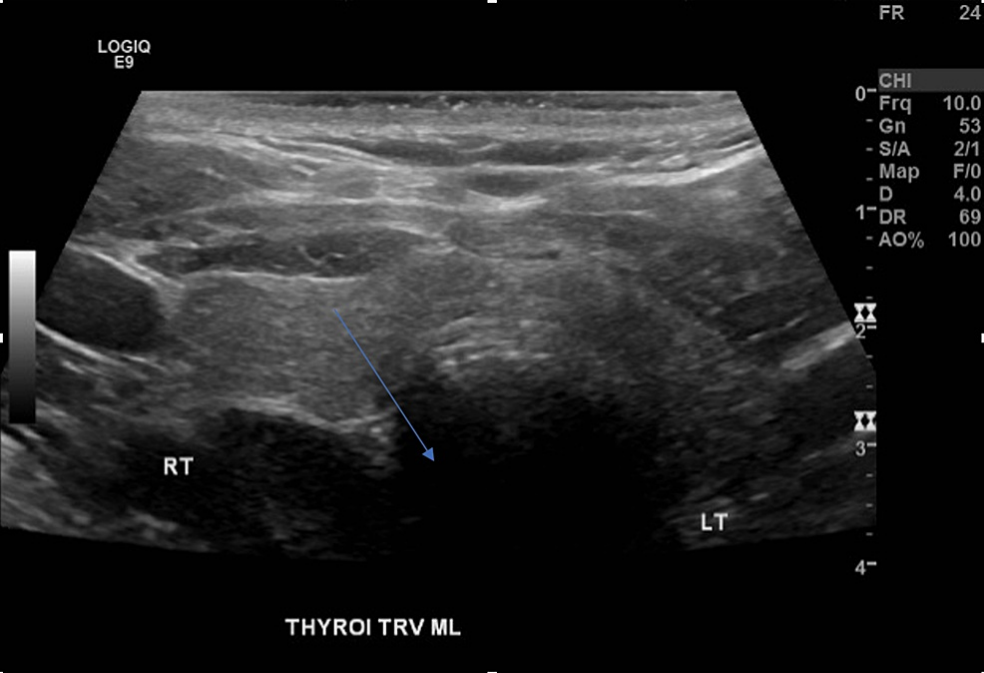
Thyroid Ultrasound

He received calcitonin (4 doses), zoledronate, aggressive hydration with IV fluids, and 2 rounds of hemodialysis. A thyroid ultrasound revealed a 0.7 x 0.4 x 0.5 cm colloid cyst in the upper right thyroid lobe (Figure 1).

**Figure 2:**
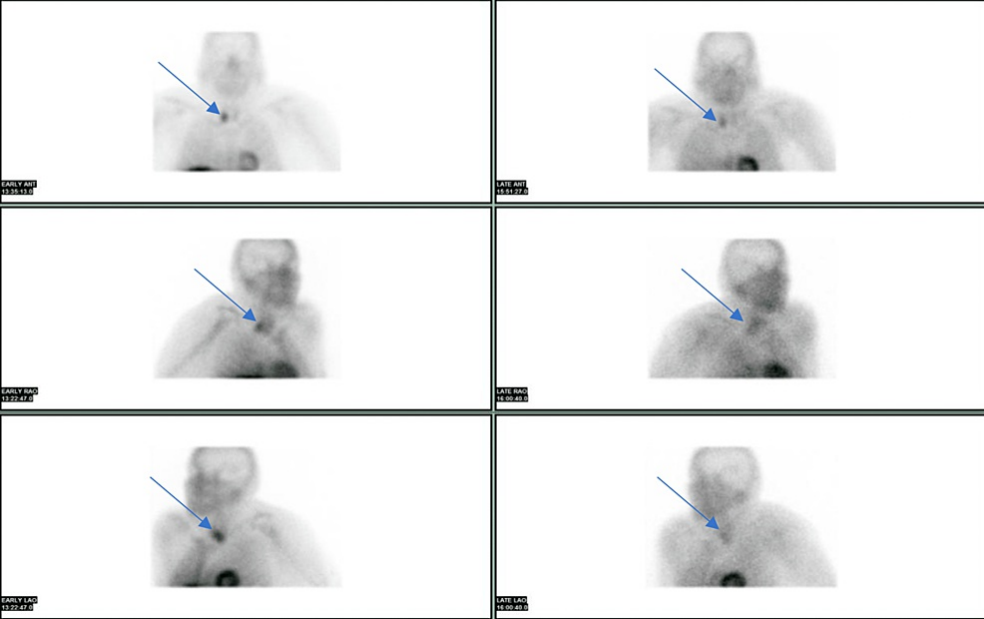
Parathyroid Nuclear Scan

The parathyroid nuclear scan showed a 26 x 17 mm presumed parathyroid adenoma in the same location. Corrected calcium dropped to 10.3, and ionized calcium dropped to 1.30 on day five, after he underwent right hemithyroidectomy and complete parathyroidectomy resulting in the removal of a large 5.3 grams and 3.2 x 2.2 x 1.5 cm parathyroid mass (Figure 2).

Immediately following the surgery, his iPTH decreased from >2,000 pg/mL to 283 pg/mL. Within 12 hours of the surgery, his iPTH was in the normal range at 87. The patient developed hungry bone syndrome given significant pre-op hypercalcemia, and treatment was initiated with 1g Tums three times per day and calcitriol 0.5 mcg daily. The final pathology of the mass revealed a parathyroid adenoma. The patient was discharged home on post-op day 11 with outpatient management.

## Discussion

Primary hyperparathyroidism is mediated through hypercalcemia, and this clinical picture includes osteitis fibrosa cystica, nephrolithiasis, and neuropsychiatric symptomatology. Severe hypercalcemia may precipitate alterations in mental status, cardiac arrhythmias, and pancreatitis [2,5,6].

Microarray-based comparative genomic hybridization and immunohistochemistry studies yielded a spectrum of genomic profiles that contained parathyroid adenomas and parathyroid carcinomas. A study conducted by Sulaiman et al. suggests the predominant genetic alterations in giant parathyroid adenomas include somatic MEN1 mutations, HRPT2 mutations, losses of chromosome 1p, and gains of chromosome 5 [1].

A point of contention is whether the increase in the size of the adenoma is correlated with the severity of hypercalcemia [1,4,5,7]. Our patient’s case indicates a positive correlation between size and hypercalcemia.

The pathogenesis of hypercalcemia in this disease begins with inappropriately elevated levels of a parathyroid hormone produced by the parathyroid gland. PTH induces osteoclast activity through the upregulation of RANK ligand production by osteoblasts [8]. The resultant hypercalcemia causes depression, anxiety, and mood disturbances through calcium-mediated alterations in dopamine, serotonin, and norepinephrine levels in the CNS, as well as glutamate-mediated cytotoxicity [9].

Renal manifestations most commonly include nephrolithiasis. Less commonly, acute kidney injury is seen. The role of hypercalcemia in AKI is multifaceted and includes hyposthenuria through the downregulation of aquaporin 2 channels and tubulointerstitial injury mediated by medullary calcium deposition [10]. Prerenal azotemia is elicited through renal vasoconstriction and prostaglandin E2-mediated reduction in NaCl reabsorption [10]. 

Hungry bone syndrome (HBS) is a rapid, intense, and prolonged hypocalcemia that follows parathyroidectomy. It is caused by a sudden drop in PTH levels and its impact on osteoclastic resorption. Risk factors include age >60, PTH level >1000pg/ml, and alkaline phosphatase level three times the upper limit of normal. The main goal of treatment is replacing calcium deficiency through supplementation with calcium salts and high doses of active vitamin D [11].

## Conclusions

This case highlights how a good internist can diagnose this case by knowing the symptoms associated with hypercalcemia along with the differentials causing it. Presenting symptoms and workup would lead to hypercalcemia as the cause of symptoms but discovering a giant Parathyroid Adenoma is a rare educational opportunity for the treating team and patient. Additionally, this case provides information on hungry bone syndrome and its treatment.
